# Prednisolone suppresses the immunostimulatory effects of 27-hydroxycholesterol

**DOI:** 10.3892/etm.2020.8458

**Published:** 2020-01-16

**Authors:** Bo-Young Kim, Yonghae Son, Min Su Kim, Koanhoi Kim

**Affiliations:** 1 Department of Pharmacology, Pusan National University School of Medicine, Yangsan, Gyeongnam 50612, Republic of Korea; 2 Department of Thoracic and Cardiovascular Surgery, Pusan National University Hospital, Seo-gu, Busan 49241, Republic of Korea

**Keywords:** chemokine ligand 2, cluster of differentiation 14, 27-hydroxycholesterol, macrophage 1 and 2 markers, prednisolone

## Abstract

In cholesterol-fed rabbits, site-specific targeting of prednisolone nanoparticles results in significantly reduced neo-intimal inflammation with a decreased infiltration of monocytes/macrophages. To understand the molecular mechanisms underlying this, the current study investigated whether prednisolone affects the immune attributes of 27-hydroxycholesterol (27OHChol), the major oxidized cholesterol molecule in circulation and tissue, in human (THP-1) monocyte/macrophage cells. THP-1 cells were exposed to 27OHChol in the presence of prednisolone followed by evaluation of inflammatory molecules at mRNA and protein levels by quantitative PCR, western blotting, ELISA and flow cytometry. The results revealed that prednisolone suppressed the 27OHChol-mediated expression of various macrophage (M)1 markers, including chemokine ligand 2, C-X-C chemokine motif 10, tumor necrosis factor-α and CD80. Treatment also impaired the 27OHCHol-enhanced migration of monocytic cells, downregulated the 27OHChol-induced cell surface expression of CD14 and inhibited the release of soluble CD14 comparable with a weakened lipopolysaccharide response. Furthermore, prednisolone suppressed the 27OHChol-induced expression of matrix metalloproteinase 9 at the transcriptional and protein level, as well as the phosphorylation of the p65 subunit. Prednisolone increased the transcription of CD163 and CD206 genes, and augmented the 27OHChol-induced transcription of CD163 without upregulating the 27OHChol-induced surface protein level of the gene. The results indicated that prednisolone inhibited the polarization of monocytes/macrophages towards the M1 phenotype, which that the immunostimulatory effects of 27OHCHol were being regulated and the immune responses in conditions that were rich in oxygenated cholesterol molecules were being modulated.

## Introduction

Prednisolone is a corticosteroid drug, predominantly comprising glucocorticoid, and is a widely used therapeutic for immune suppression (1). The drug abrogates the expression of inflammatory genes by inhibiting the transcriptional promoting activity of the AP-1 and NF-κB transcription factors, and also enhances the release of anti-inflammatory proteins such as IL-4, IL-13 or IL-10 (2,3), suggesting that prednisolone modulates tissue responses by regulating gene regulation at sites of inflammation (4). Data of animal studies suggest that prednisolone, the active metabolite of prednisone, exerts its pharmacological effects in cholesterol-rich environments. Prednisone, which is metabolized by the liver to prednisolone, inhibits development of inflammatory lesions in the aortas of cholesterol-fed rabbits, without lowering serum cholesterol levels (5). Site-specific targeting of nanoparticles of prednisolone reduces inflammation and formation of neo-intima in a high cholesterol diet rabbit model of established atheroma (6). However, it is yet to be established how prednisolone affects tissue responses occurring in a milieu rich in cholesterol molecules.

Oxysterols, the oxygenated derivatives of cholesterol, induce expression of inflammatory molecules by monocytes/macrophages and are recognized as strong inducers of inflammation (7–9). 27-Hydroxycholesterol (27OHChol) is the most abundant oxysterol in circulation and tissues under hypercholesterolemic conditions (10). 27OHChol promotes migration of monocytic cells and T lymphocytes expressing CCR5 (11,12), enhances the production of molecules involved in various inflammatory processes, including TNF-α and CXCL8 (13–15), and increases the expression of MHC I and II molecules and pattern recognition receptors, thereby augmenting responses of immune cells to pathogen-associated molecular patterns (16–18). These findings suggest that 27OHChol steers macrophages/monocytes to an immunostimulatory phenotype.

As the key innate immune effector cells, macrophages are highly heterogeneous and are capable of rapidly changing their functions in response to local microenvironmental signals (19). The activated macrophages (M1) driven by interferon-γ and lipopolysaccharide (LPS) release inflammatory and immunostimulatory cytokines (20). The alternatively activated macrophages (M2) are elicited by IL-4, immune complexes, or glucocorticoids, in combination or not with transforming growth factor-β, and act to restrict these inflammatory responses through secretion of immunoregulatory cytokines (20), thereby affecting angiogenesis and invasiveness (21). Controlling the macrophage polarization results in altered disease progression, indicating that macrophage polarization serves as a novel therapeutic approach.

In the present study, we used human THP-1 monocytic cells to examine whether prednisolone modifies the 27OHChol-mediated polarization and responses of monocytes/macrophages. Dexamethasone was employed as a positive control because it is a potent, long-acting steroid product and is reported to modify cellular responses to oxysterol (22,23). This study determines the new biological activities of prednisolone that contribute to pharmacological effects of the drug.

## Materials and methods

### 

#### Cells and reagents

The human THP-1 monocyte/macrophage cell line was purchased from the American Type Culture Collection (ATCC) and maintained as suggested by the ATCC. Prednisolone and LPS were purchased from Sigma-Aldrich and InvivoGen, respectively. 27OHChol and antibodies against CD14, p65, phosphorylated p65, and β-actin were obtained from Santa Cruz Biotechnology Inc.

#### Reverse transcription (RT) - polymerase chain reaction (PCR)

Total RNA was reverse-transcribed for 1 h at 42°C using the Moloney murine leukemia virus reverse transcriptase, and real-time quantitative PCR was performed in triplicate using a LightCycler^®^ 96 Real-Time PCR System (Roche), as previously reported (24). Each 20 µl reaction mixture consisted of 10 µl of SYBR Green Master Mix and 2 µl each of 10 pM forward and reverse primers of the gene to be quantified. The thermal cycling conditions were as follows: 95°C for 10 min, followed by 45 cycles at 95°C for 10 sec, 50°C for 10 sec, and 72°C for 10 sec. The LightCycler^®^ 96 software (v1.1.0.1320; Roche) was applied to calculate the relative expression of each gene as the ratio to the housekeeping gene, glyceraldehyde 3-phosphate dehydrogenase (GAPDH). Target gene mRNA levels were normalized to GAPDH using the 2^−ΔΔCt^ method (25). The primers used for real-time PCR were as follows: TNF-α, 5′-CCCAGGGACCTCTCTCTAATC-3′ (forward) and 5′-ATGGGCTACAGGCTTGTCACT-3′ (reverse); IL-1β, 5′-TGAGCTCGCCAGTGAAATGA (forward) and 5′-AGATTCGTAGCTGGATGCCG-3′ (reverse); CXCL10, 5′-TGTACGCTGTACCTGCATCA-3′ (forward) and 5′-GGACAAAATTGGCTTGCAGGA-3′ (reverse); CXCL11, 5′-AAGCAGTGAAAGTGGCAGAT-3′ (forward) and 5′-TAAGCCTTGCTTGCTTCGAT-3′ (reverse); CD80, 5′-GCAGGGAACATCACCATCCA-3′ (forward) and 5′-TCACGTGGATAACACCTGAACA-3′ (reverse); CD86, 5′-GGACTAGCACAGACACACGGA-3′ (forward) and 5′-CTTCAGAGGAGCAGCACCAGA-3′ (reverse); CD163, 5′-AAAAAGCCACAACAGGTCGC-3′ (forward) and 5′-CTTGAGGAAACTGCAAGCCG-3′ (reverse); CD206, 5′-TGAATTGTACTGGTCTGTCCT-3′ (forward) and 5′-CTGTGGTGCTGTGCATTTATCT-3′ (reverse); CCL2, 5′-CAGCCAGATGCAATCAATGCC-3′ (forward) and 5′-TGGAATCCTGAACCCACTTCT-3′ (reverse); matrix metalloprotease-9 (MMP-9), 5′-GCACGACGTCTTCCAGTACC-3′ (forward) and 5′-CAGGATGTCATAGGTCACGTAGC-3′ (reverse); CD14, 5′-ACGCCAGAACCTTGTGAGC-3′ (forward) and 5′-GCATGGATCTCCACCTCTACTG-3′ (reverse); and GAPDH, 5′-GAAGGTGAAGGTCGGAGT-3′ (forward) and 5′-GAAGATGGTGATGGGATTTC-3′ (reverse).

#### Chemotaxis assay

Cell migration was measured using Transwell Permeable Supports (Costar) as previously described (12). Briefly, 5×10^5^ cells in 100 µl 0.1% BSA were loaded into the top chamber of 5-µm-pore polycarbonate transwell inserts. Transwell chambers were inserted into wells filled with supernatant harvested from THP-1 cells treated with 27OHChol, with or without prednisolone. After incubation for 3 h at 37°C, the number of cells that migrate to the bottom chamber was counted using a Vi-Cell cell counter (Beckman Coulter, Inc.).

#### MMP-9 gelatinolytic activity in supernatants

Supernatants isolated from THP-1 cells were collected and concentrated 30-fold using Vivaspin 2 Centricon, as previously described (26). The concentrated medium was then electrophoretically separated onto an 8% polyacrylamide gel containing 0.15% gelatin. After electrophoresis, the gel was washed, activated, and stained with 0.2% Coomassie brilliant blue R-250, followed by destaining. Clear zones against the blue background indicate gelatinolytic activity.

#### Flow cytometric analysis

Surface levels of CD molecules were evaluated as previously reported (18). Briefly, THP-1 cells were harvested by centrifugation, followed by incubation for 40 min at 4°C with anti-CD14 antibody conjugated with a green fluorescent dye (Santa Cruz Biotechnology Inc.), FITC anti-human CD163 and PE anti-human CD206 (BioLegend). After washing twice with phosphate-buffered saline (PBS), cells were re-suspended in 1% paraformaldehyde in PBS. Fluorescence was analyzed by flow cytometry.

#### Enzyme-linked immunosorbent assay

The levels of CCL2, sCD14, and MMP-9 secreted into the culture media were determined using commercially available enzyme-linked immunosorbent assay (ELISA) kits as per the manufacturer's instructions (R&D Systems), following the previously described protocols (17).

#### Western blot analysis

Cell lysates were separated by 10% SDS-PAGE and subsequently transferred to nitrocellulose membranes. After blocking for 1 h in 1% skim milk (prepared in TBS containing 0.05% Tween-20), membranes were incubated overnight at 4°C, with primary antibodies against CD14, phosphorylated p65, p65 subunit, or β-actin. After three washes with TBS-T, the membranes were incubated for 1 h with HRP-conjugated secondary antibodies at room temperature. Bands were detected using chemiluminescent detection reagents.

#### Statistical analysis

One-way ANOVA followed by Tukey's multiple comparison tests were performed using PRISM (version 5.0) (GraphPad Software Inc.). Data are presented as the mean ± SD, and are representative of three independent experiments. Null hypotheses of no difference were rejected for P-values less than 0.05.

## Results

### 

#### Prednisolone inhibits the expression of M1 markers in monocytes/macrophages

**Figure 1.**
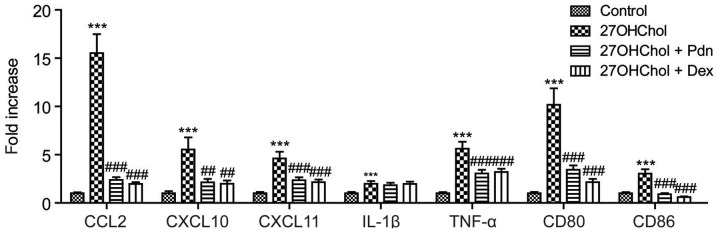
Inhibited expression of 27OHChol-induced M1 markers. THP-1 cells (2.5×10^5^ cells/ml) were serum-starved overnight and cultured for 48 h with 27OHChol (6.2 µM) in the presence of 1 µM Pdn or Dex. Transcript levels of the indicated M1 markers were assessed via reverse transcription-quantitative PCR. Data are presented as the mean ± standard deviation (n=3). ***P<0.001 vs. control; ^###^P<0.001 vs. 27OHChol; ^##^P<0.01 vs. 27OHChol. 27OHChol, 27-hydroxycholesterol; CCL2, chemokine ligand 2; CXCL, C-X-C chemokine motif 10; TNF-α, tumor necrosis factor-α; IL, interleukin; Pdn, prednisolone; Dex, dexamethasone.

We determined whether prednisolone affects the expression of the M1 phenotype markers (Fig. 1). Stimulation of monocyte/macrophage cells with 27OHChol results in increased expression of molecules associated with the M1 phenotype, such as CCL2, CXCL10, CXCL11, IL-1β, TNF-α, CD80 and CD86, but the 27OHChol-induced expression of M1 markers (except IL-1β) is significantly suppressed following exposure to prednisolone. The inhibition of M1 marker expressions is similar to that exerted by dexamethasone, which is used as a positive control due to its potent, long-acting activity. These results indicate that prednisolone regulates polarization to the M1 phenotype.

#### Prednisolone impairs migration of monocytic cells via inhibiting CCL2 expression

**Figure 2.**
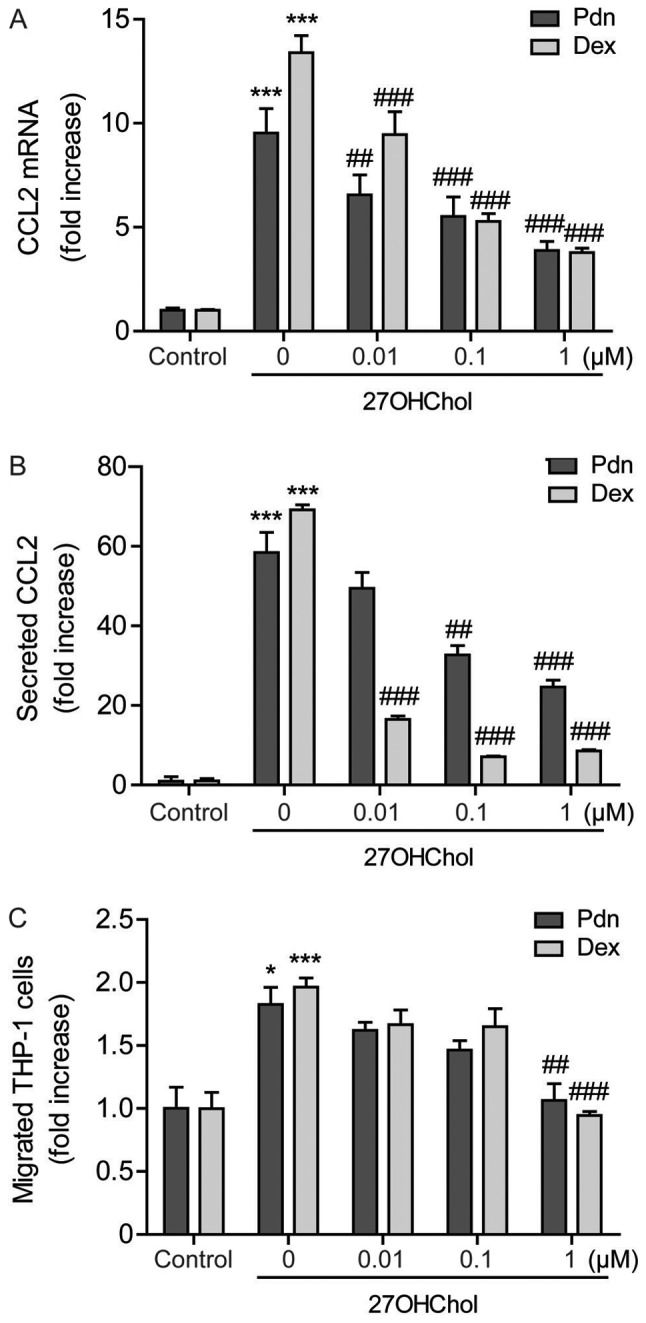
Impaired CCL2 expression and monocytic cell migration. Serum-starved THP-1 cells (2.5×10^5^ cells/ml) were cultured for 48 h with 27OHChol in the presence of varying concentrations of Pdn or Dex. (A) Levels of CCL2 transcript were assessed via reverse transcription-quantitative PCR. values are provided as mRNA levels normalized to GAPDH expression, relative to that of the non-treated cells (control). (B) Culture media were isolated, and CCL2 protein levels in the media were determined via ELISA. (C) Monocytic cells were exposed to conditioned media isolated from THP-1 cells treated with 27OHChol with or without Pdn or Dex. Cell migration was measured using a chemotaxis assay. Data are presented as the mean ± standard deviation (n=3). ***P<0.001 vs. control; *P<0.05 vs. control; ^###^P<0.001 vs. 27OHChol; ^##^P<0.01 vs. 27OHChol. CCL2, chemokine ligand 2; 27OHChol, 27-hydroxycholesterol; Pdn, prednisolone; Dex, dexamethasone.

Since CCL2 is the key M1 molecule regulating migration of monocytes/macrophages, we examined the effects of prednisolone, in parallel with dexamethasone, on CCL2 expression at the transcriptional and protein levels. The 27OHChol-induced transcription of CCL2 is attenuated in a dose-dependent manner after treatment with prednisolone, and which is comparable to that obtained with dexamethasone (Fig. 2A). Exposure to prednisolone also significantly reduces the amount of secreted CCL2. Of the two steroids, CCL2 is reduced to a greater extent after exposure to dexamethasone, indicating that dexamethasone is more efficacious in inhibiting the CCL2 secretion (Fig. 2B). We further determined the influence of prednisolone on cell migration. Significant increase of monocytic cell migration is observed in response to the supernatants harvested following stimulation with 27OHChol. The cell migration is reduced when cells are exposed to supernatants isolated from cells cultured with 27OHChol plus 1 µM prednisolone or dexamethasone. The reduction caused by prednisolone is comparable to that obtained by dexamethasone (Fig. 2C). These results indicate the impairment of 27OHChol-induced CCL2 expression and cell migration following exposure to prednisolone.

#### Prednisolone down-regulates 27OHChol-induced CD14 expression and weakens LPS response

**Figure 3.**
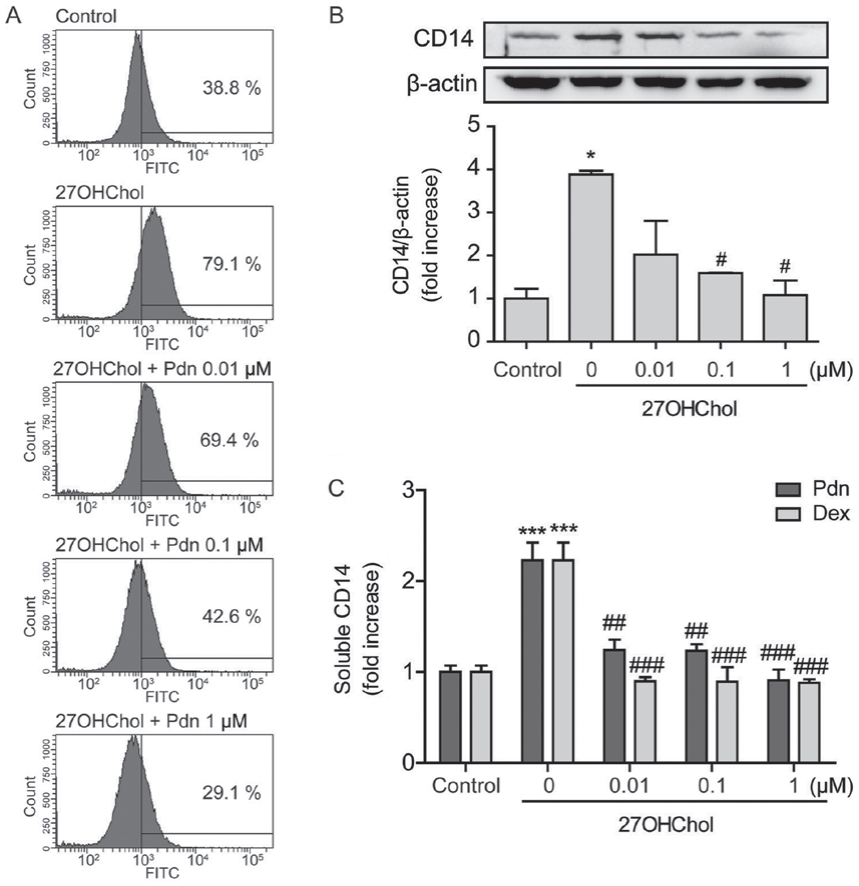
Downregulation of CD14 protein. THP-1 cells (2.5×10^5^ cells/ml) were serum-starved and cultured for 48 h with 27OHChol in the presence of the indicated concentrations of Pdn or Dex. (A) Cells were immunostained with surface CD14 and analyzed using flow cytometry. (B) Cell extracts were obtained after treatment with or without 27OHChol and Pdn, followed by Western blot analysis to detect CD14 and β-actin. CD14 protein expression was quantified via densitometry. Data are expressed as the mean ± standard deviation of three independent experiments. (C) Culture media were harvested, and the amount of CD14 protein secreted into the media was measured by ELISA. Data are expressed as the means ± SD of three independent experiments. *P<0.05 vs. control; ^#^P<0.05 vs. 27OHChol; ***P<0.001 vs. control; ^###^P<0.001 vs. 27OHChol; ^##^P<0.01 vs. 27OHChol. 27OHChol, 27-hydroxycholesterol; Pdn, prednisolone; Dex, dexamethasone.

We next investigated whether prednisolone influences the expression of CD14. Stimulation with 27OHChol results in upregulation of CD14 on the monocyte/macrophage cell surface, as indicated by the increased percentage of CD14-positive cells, but the percentage decreases in a dose-dependent manner after treatment with prednisolone (Fig. 3A). The levels of CD14 protein were also evaluated using Western blot analysis. 27OHChol exposure increases the levels of cellular CD14 protein, which reduce to the basal level and lower, following treatment with 0.1 and 1 µM of prednisolone, respectively (Fig. 3B). However, we were unable to obtain conclusive data that prednisolone affects the levels of CD14 transcripts (Fig. S1). We further investigated the effects of prednisolone on secretion of soluble CD14 (sCD14). The 27OHChol-induced sCD14 release is almost completely inhibited after treatment with prednisolone, which is comparable to results obtained with dexamethasone (Fig. 3C). These results indicate that prednisolone affects CD14 expression at the protein level.

The effects of prednisolone were also determined on LPS stimulation, by measuring the CCL2 expression (Fig. S2). Levels of CCL2 transcripts were observed to increase 33.8- and 11.1-folds after stimulation with 27OHChol and LPS, respectively. Addition of LPS to 27OHChol-exposed cells resulted in further elevation of CCL2 transcripts by 115.4- fold, which reduces to 79.2-, 29.8- and 9.8-folds in the presence of 0.01, 0.1 and 1 µM of prednisolone, respectively. Collectively, our data indicate that prednisolone down-regulates CD14 and thereby inhibits the 27OHChol-enhanced LPS response.

#### Prednisolone inhibits 27OHChol-induced MMP-9 expression

**Figure 4.**
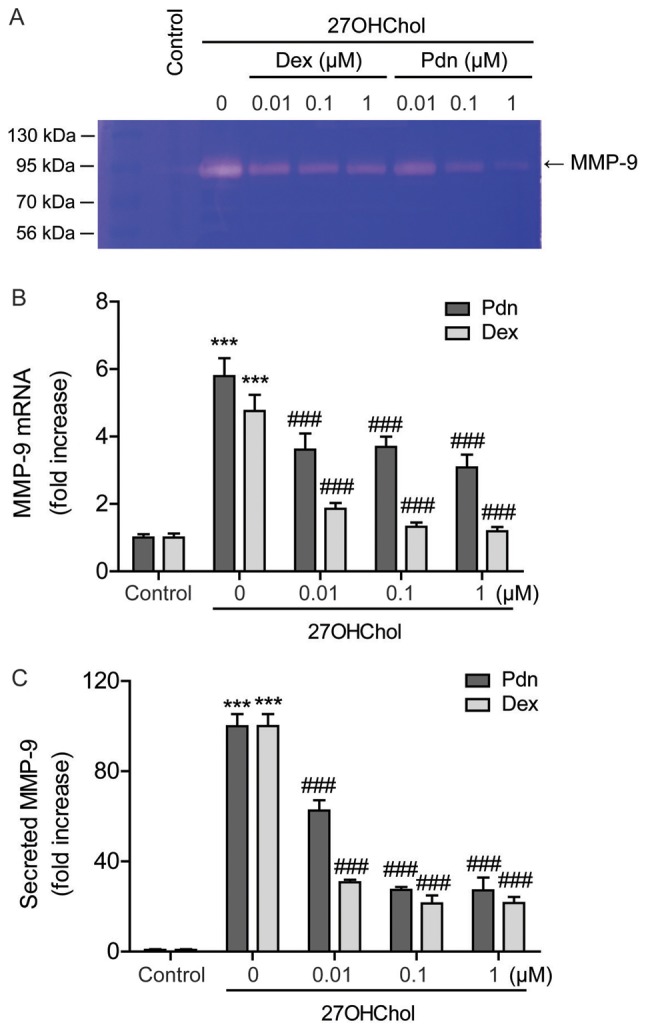
Decreased 27OHChol-induced MMP-9 production. Serum-starved THP-1 cells (2.5×10^5^ cells/ml) were cultured with 27OHChol in the presence of the indicated concentrations of Pdn or Dex for 48 h. (A) The activity of MMP-9 secreted by cells was assessed by gelatin zymography. (B) Levels of MMP-9 transcript were assessed via reverse transcription-quantitative PCR. (C) Culture media was isolated and the levels of MMP-9 in the media were measured via ELISA. Data are expressed as the mean ± standard deviation (n=3). ***P<0.001 vs. control; ^###^P<0.001 vs. 27OHChol. 27OHChol, 27-hydroxycholesterol; Pdn, prednisolone; Dex, dexamethasone; MMP9, matrix metallopeptidase 9.

Since prednisolone inhibits the sCD14 release, we evaluated the effects of the drug on MMP-9 activity. 27OHChol enhances MMP-9 activity in the cell supernatant, which decreases after exposure to prednisolone, as demonstrated by gelatin zymography (Fig. 4A). Furthermore, evaluating the effects of prednisolone on MMP-9 expression reveals elevated levels of MMP-9 transcripts after 27OHChol exposure, which is suppressed by treatment with prednisolone (Fig. 4B). The 27OHChol-induced MMP-9 secretion is also significantly suppressed following treatment with prednisolone, as determined by ELISA (Fig. 4C). Compared to prednisolone, dexamethasone inhibits the transcription and secretion of MMP-9 with higher-potency (Fig. 4B and C). These results indicate that although less effective than dexamethasone, prednisolone inhibits MMP-9 expression at the transcriptional and protein levels.

#### Prednisolone regulates molecular signaling enhanced by 27OHChol

**Figure 5.**
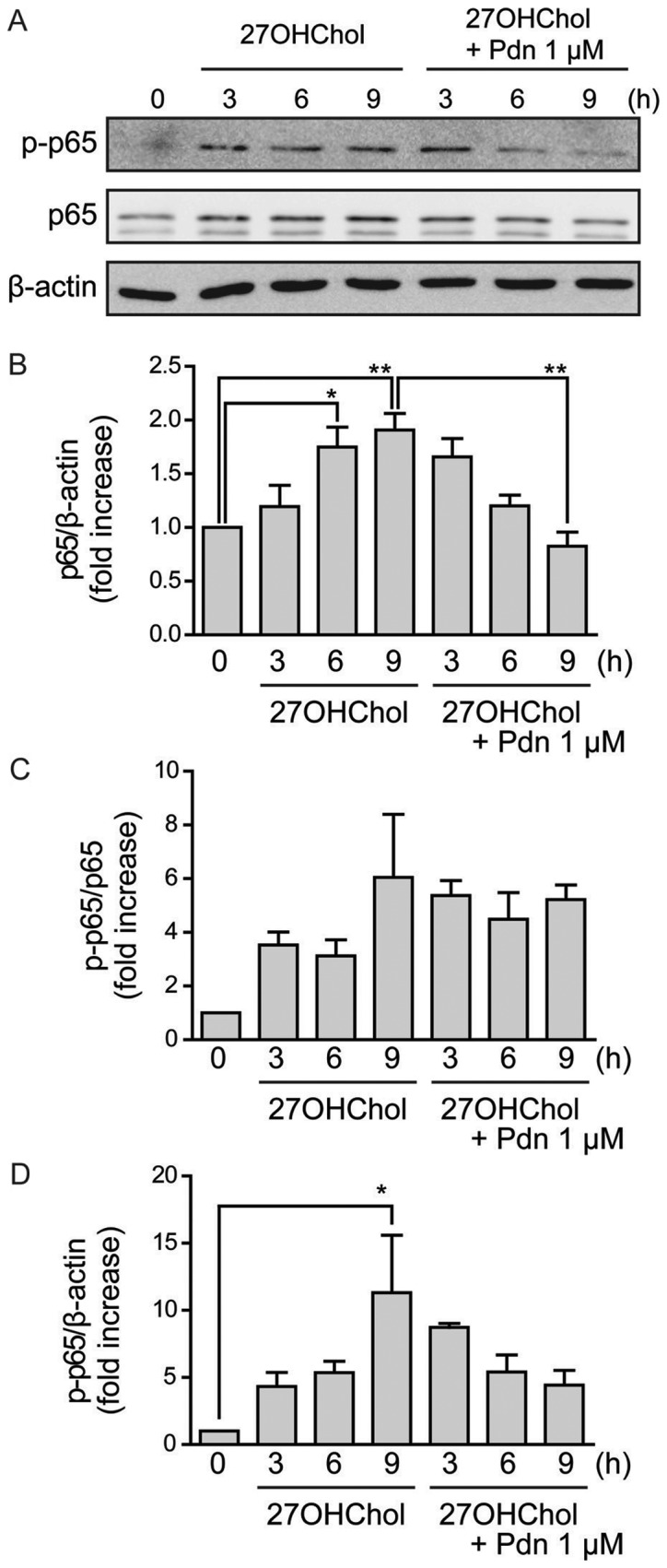
Suppression of 27OHChol-induced p65 subunit phosphorylation after exposure to prednisolone. Following overnight serum-starvation, THP-1 cells were exposed for the indicated time periods to 27OHChol (6.2 µM) in the absence or presence of 1 µM Pdn. (A) Whole cell extracts were isolated and subjected to immunoblotting for p65, p-p65 and β-actin. Data are representative of three independent experiments. The relative expression of (B) p65/β-actin (C) p-p65/p65 and (D) p-p65/β-actin are presented. Data are expressed as the mean ± standard deviation of three independent experiments. *P<0.05 and **P<0.01 as indicated. 27OHChol, 27-hydroxycholesterol; Pdn, prednisolone; p, phosphorylated.

We investigated the effects of prednisolone on expression levels of the NF-κB p65 subunit and its phosphorylated form by performing Western blot analyses. 27OHChol increases the levels of p65 subunit, which is suppressed following treatment with prednisolone (Fig. 5A and B). The phosphorylated form of p65 (p-p65) may only elevate because total p65 expression increases, rather than increased p65 phosphorylation (Fig. 5C and D). These results suggest that prednisolone suppresses the 27OHChol-induced activity of the transcription factor NF-κB.

#### Prednisolone affects transcript levels of CD163 and CD206

**Figure 6.**
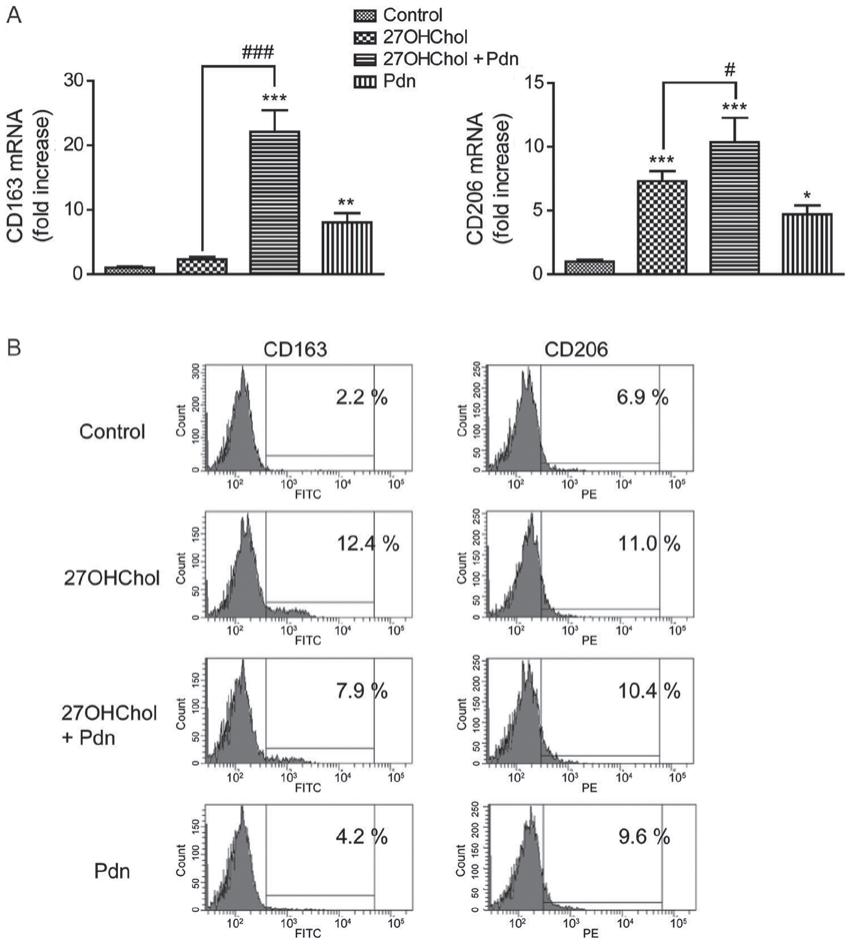
Differential effects of prednisolone on the expression of CD163 and CD206. After overnight serum-starvation, THP-1 cells were treated with 27OHChol for 48 h in the absence or presence of Pdn. (A) Transcript levels of CD163 and CD206 were assessed using reverse transcription-quantitative PCR. Data are expressed as the mean ± standard deviation (n=3). ***P<0.001 vs. control; **P<0.01 vs. control; *P<0.05 vs. control; ^###^P<0.001 vs. 27OHChol; ^#^P<0.05 vs. 27OHChol. (B) Cells were immunostained for surface CD163 and CD206, and subsequently analyzed via flow cytometry. Data are representative of three independent experiments. 27OHChol, 27-hydroxycholesterol; Pdn, prednisolone.

We subsequently examined the effects of prednisolone on the 27OHChol-induced expression of M2 markers (Fig. 6). Prednisolone exposure increases the transcript levels of CD163 and augments the 27OHChol-induced transcription of the CD163 gene. However, although an increase is observed in the transcript levels of CD206, there was no amplification in the 27OHChol-induced transcription of CD206 gene; the transcript levels of CD206 following cotreatment with 27OHChol and prednisolone were comparable to the summation of the levels observed with each treatment alone (Fig. 6A). We further determined whether prednisolone affects the expression levels of CD163 and CD206 molecules on the cell surface (Fig. 6B). We observed an increase in the surface levels of CD163 and CD206 after exposure to 27OHChol or prednisolone. Investigating the levels of CD68 after 27OHChol and prednisolone exposure shows no increase in the expression of CD68 (Fig. S3). Taken together, these results suggest that prednisolone differentially regulates the transcript levels and surface expression of M2 makers in the presence of 27OHChol.

## Discussion

27OHChol enhances the expression of anti-inflammatory and inflammatory molecules, and cytokines and chemokines of monocytic cells (12,27,28), indicating that 27OHChol is involved in the polarization of monocytes/macrophages. However, the effects of 27OHChol on M1/M2 polarization have been elusive. We therefore investigated the expressions of M1 and M2 markers, to understand the overall influence of 27OHChol on monocyte/macrophage polarization. Increases in M1 markers validate that 27OHChol is an active molecule with inflammatory functions, whereas the increased transcription of M2 markers (such as CD163 and CD206) (24) is in agreement with a previous study that reported polarization of human macrophages toward the M2 immunomodulatory phenotype after short-term exposure to this oxysterol (27). These findings suggest that 27OHChol affects both M1 and M2 polarization of monocytes/macrophages. Besides, 27OHChol does not change CD68 expression, which agrees with the fact that CD68 is expressed both in M1 and M2 macrophages (29), and indicates that 27OHChol is unlikely to cause differentiation or polarization of monocytic cells into other lineages because CD68 can be used as a pan-macrophage marker. Of the two markers, expression of M1 markers is more strongly enhanced following treatment with 27OHChol. The preferential expression of M1 markers could help explain the dominance of immunostimulatory and inflammatory responses in a milieu rich in 27OHChol in spite of its liver X receptor agonistic activity which suppresses inflammatory signaling in macrophages (30,31).

We next attempted to determine the effects of prednisolone on M1/M2 polarization under hypercholesterolemic conditions. We observed suppressed expression of 27OHChol-induced M1 markers, and upregulation of the transcription and cell surface expression of CD163 and CD206, without further enhancement of the 27OHChol-induced expression of molecules. These results are consistent with previous reports that glucocorticoids generate M2 macrophages (20) and enhance transdifferentiation of macrophages towards the immune regulatory M2 phenotype (32). Taken together with previous publications, our findings suggest that prednisolone differentially regulates M1/M2 polarization of monocytes/macrophages. The differential effects are likely to contribute to the immune suppressive activity of the drugs in 27OHChol-rich conditions.

Prednisolone and dexamethasone exhibit not only high effectiveness but also differences in their potency with respect to suppression of 27OHChol-mediated immune stimulation. Dexamethasone more effectively inhibits secretion of CCL2 and transcription of MMP-9, than prednisolone. We believe that the differences in the inhibitory activity are in line with pharmacokinetics and pharmacological activity of the drugs. Prednisolone is an intermediate acting steroid with a half-life of 12 to 36 h, whereas dexamethasone is a long acting corticosteroid with a biological half-life between 36 and 72 h; furthermore, dexamethasone is five to six times as potent as prednisolone in terms of anti-inflammatory potential (23,33).

NF-κB is one of the most important regulators of pro-inflammatory gene expressions such as TNF-α, IL-1β, and IL-6 (34); also, the activation of macrophages in response to multiple M1 polarizing stimuli is regulated primarily via NF-κB (35,36). Therefore, we investigated the possible involvement of NF-κB in 27OHChol-induced M1 polarization. Increased expression of M1 markers coincides with enhanced phosphorylation of the p65 subunit of NF-κB following 27OHChol treatment, and prednisolone suppresses both the expression of M1 markers and phosphorylation of p65, without affecting M2 markers. These results suggest a correlation between activity of inducible NF-κB and regulation of M1 polarization in the presence of 27OHChol and prednisolone.

During an inflammatory response, the expression and secretion of MMP-9 is elevated by macrophages, and its activity is required for migration of macrophages (37). CCL2 is a key molecule recruiting monocytes to the sites of inflammation (38). Prednisolone not only suppresses MMP-9 expression but also decreases monocytic cell migration coupled with CCL2 production. These results are in line with the findings by Wong *et al*, who reported decreased MMP-9 expression in macrophages and reduced infiltration of inflammatory cells following treatment with prednisolone (39). MMP-9 is also involved in the post-translational processing of CD14. Proteolytic cleavage of mCD14 by MMP-9 results in sCD14 shedding (40). CD14 binds to LPS, and the LPS-CD14 complex triggers macrophage activation, culminating in inflammatory responses by enhancing the production of multiple inflammatory molecules (41). Our studies determined that prednisolone down-regulates CD14 and attenuates the LPS response. Taken together, these results indicate that MMP-9 may be one of the key molecules that mediate the 27OHChol-induced inflammatory and immune responses.

This study reports a new pharmacological effect of prednisolone: The differential regulation of M1 and M2 markers in a milieu rich in 27OHChol. We believe that differential regulation of M1/M2 polarization of monocyte/macrophage cells is a promising strategy for suppression of the immune reactions activated due to cholesterol oxidation products.

## Supplementary Material

Supporting Data

## Data Availability

The datasets used and/or analyzed during the present study are available from the corresponding author on reasonable request.
