# Supplementary material for: Prednisolone suppresses the immunostimulatory effects of 27-hydroxycholesterol
Source: Exp Ther Med. 2020 Jan 16;19(3):2335–42. doi: 10.3892/etm.2020.8458 (PMC7041177; doi:10.3892/etm.2020.8458)

Figure S1. Effects of Pdn on levels of CD14 transcripts. After serum starvation, THP-1 cells were treated for 48 h with indicated amount of Pdn in the presence of 27OHChol. Levels of CD14 transcripts were assessed by quantitative PCR. The y-axis values represent the increases of CD14 mRNA levels normalized to GAPDH levels, relative to that of the non-treated THP-1 cells (control). Data are presented as the mean ± SD (n=3 replicates for each group). ***P<0.001 vs. control. Pdn, prednisolone.

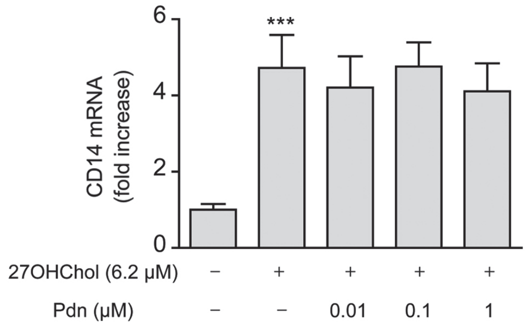

Figure S2. Effects of Pdn on CCL2 production in response to LPS in the presence of 27OHChol. Serum-starved THP-1 cells were cultured for 24 h with 27OHChol in the presence of Pdn, followed by stimulation for 9 h with or without LPS. Levels of CCL2 transcripts were assessed by quantitatie PCR. Data are presented as the mean ± SD (n=3 replicates for each group). *P<0.05 vs. control; +++P<0.001 vs. 27OHChol; ##P<0.01 vs. 27OHChol+LPS; ###P<0.001 vs. 27OHChol+LPS. Pdn, prednisolone; LPS, lipopolysaccharide; CCL2, chemokine ligand 2.

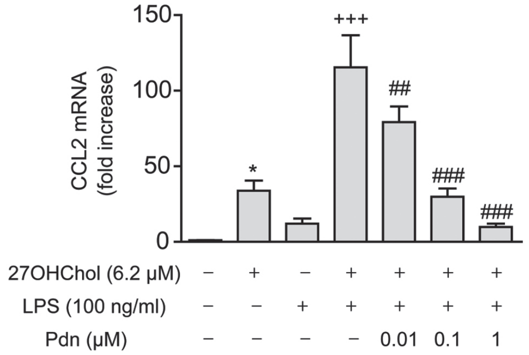

Figure S3. Effects of 27OHChol and Pdn on CD68 expression. Serum-starved THP-1 cells were treated for 48 h with 27OHChol in the absence or presence of Pdn. (A) Transcripts of CD68 were amplified by reverse transcription PCR. (B) Relative CD68 band intensity was semi-quantified by normalizing to β-actin. Data are presented as the mean ± SD (n=3 replicates for each group). Pdn, prednisolone.

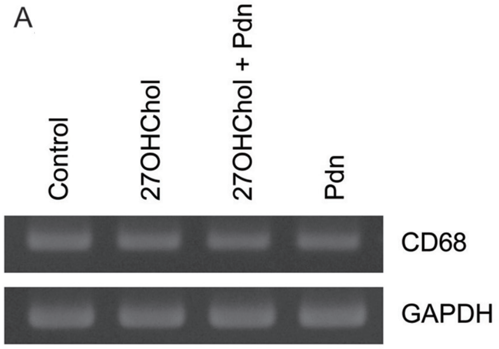

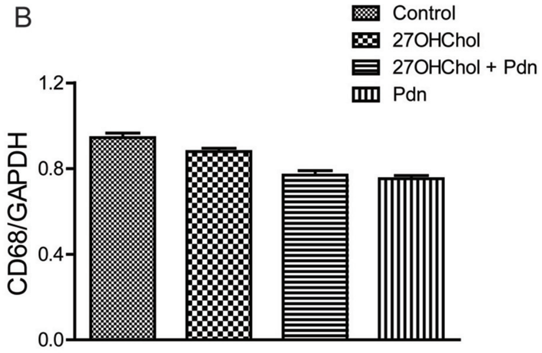

Supplement: Supporting Data [file Supplementary_Data.pdf]
